# Human papillomavirus oncoproteins and apoptosis (Review)

**DOI:** 10.3892/etm.2013.1374

**Published:** 2013-10-30

**Authors:** PEIYUE JIANG, YING YUE

**Affiliations:** Department of Gynecological Oncology, First Hospital of Jilin University, Changchun, Jilin 130021, P.R. China

**Keywords:** human papillomavirus, oncoprotein, incorporation, apoptosis

## Abstract

The aim of this study was to review the literature and identify the association between human papillomavirus (HPV) oncoproteins and apoptosis. HPV-associated apoptosis may be primarily blocked by a number of oncoproteins, including E5, E6 and E7. E5 protein protects cells from tumor necrosis factor-associated apoptosis; the oncoprotein E6 predominantly inhibits apoptosis through the p53 pathway; and oncoprotein E7 is involved in apoptosis activation and inhibition. In addition, HPV oncoproteins are involved in activating or repressing the transcription of E6/E7. In conclusion, HPV oncoproteins, including E5, E6 and E7 protein, may interfere with apoptosis via certain regulatory principles.

## 1. Introduction

Human papillomaviruses (HPVs) are small, double-stranded DNA viruses. HPV infection occurs on the surface of the epithelium in the basal layer, and the life cycle of the virus is distinguished according to the differentiation program of the infected cells. The HPV family encompasses ~200 categories (1), of which 40 categories have been isolated from the genital tract (2). On the basis of HPV clinical associations, HPVs may be divided into two types: high- or low-risk. High-risk HPVs participate in the development of cervical neoplasia in persistently infected females (3). Of these, HPV-16, -18, -31 and -35 are the most prevalent, and are commonly associated with lesions that may progress to high-grade intraepithelial neoplasia and ultimately to carcinoma. The other types of HPV, including HPV-6 and -11, are low-risk and have been shown to be predominantly associated with benign lesions, which seldom progress to cancer (4). HPV DNA has been detected in <99.7% of all cases of cervical cancer. Moreover, infection with HPV-16 and -18 accounts for >50% of all cervical cancer (5), and cervical cancer accounts for one-fifth of all cancer-associated mortalities among females diagnosed each year, making it the second most common type of cancer in females worldwide (6). In addition, a number of low-risk types may be transmitted sexually and may cause genital condyloma (7). All types of HPV share a common genomic structure and encode eight proteins, including six early proteins: E1, E2, E4, E5, E6 and E7, and two late proteins. In particular, E5, E6 and E7 oncoproteins of the high-risk strains are considered to be antiapoptotic oncoproteins, and the main contributors to malignant transformation (8). E2 and E7 are also considered to be proapoptotic proteins. Therefore, there is a close correlation between apoptosis and the regulation of the three oncoproteins, E5, E6 and E7.

## 2. E6 oncoprotein

**Figure 1:**
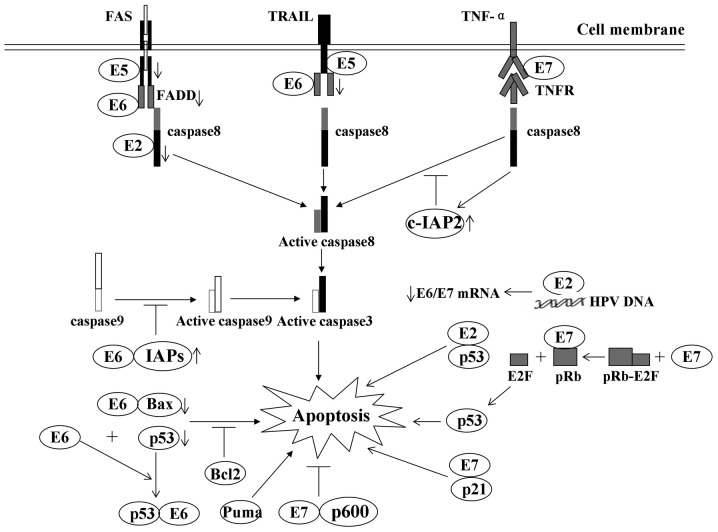
Modulation of apoptosis by HPV early proteins: A number of the interactions of viral oncoproteins with cellular proteins and their effects on apoptosis. E2 protein is able to induce apoptosis by downregulating the transcription of E6/E7 mRNA and by binding with p53, respectively. E5 impairs the formation of the death-inducing signaling complex triggered by FasL and TRAIL. E6 protein inhibits apoptosis by targeting proapoptotic proteins, including p53 and Bax, for proteolytic degradation. E6 protein also protects cells from death receptor-induced apoptosis by blocking apoptotic signal transduction and inducing the expression of IAPs. E7 oncoprotein-expressing cells are usually predisposed to undergo apoptosis, in part due to degradation of the antiapoptotic protein pRb and E2F-1 binding. Downwards arrow, degradation of cellular proteins; inhibition arrow, inhibition status of the pathway; upwards arrow, upregulation of proteins. HPV, human papillomavirus; FasL, Fas ligand; TNF, tumor necrosis factor; TRAIL, TNF-related apoptosis-inducing ligand; IAP, inhibitor of apoptosis; TNFR, TNF receptor; FADD, Fas-associated protein with death domain.

The HPV E6 oncoprotein is a relatively small protein. With regard to HPV-16, the E6 oncoprotein is comprised of 150 amino acids, containing two CX_2_C-X_29_-CX_2_C zinc-like fingers joined by an interdomain linker of 36 amino acids (9,10). For the high-risk E6 genes, the truncated E6 (encoding residues 1–41 HPV-16 E6) is able to inactivate the functions of full-length E6 by binding to the interface of the C- and N-terminal halves of the E6 protein (11,12). Notably, E6 protein has no enzymatic activities and the majority of the activities are considered to be triggered by protein-protein interactions (13). The first and most common protein that interacts with E6 is E6-associated protein (E6AP), a ubiquitin ligase (14). The ubiquitin cascade functions to target proteins for proteasomal degradation by adding multiple ubiquitin monomers to the protein destined to be destroyed. Therefore the E6, E6AP and target proteins form a complex, which leads to ubiquitination of the target protein and subsequent proteasome-mediated degradation (15). One of the main targets of E6 protein is the tumor suppressor p53, which is a DNA site-specific transcription factor and one of the key signaling coordinators in the cell following genotoxic or cytotoxic stress (16). The E6 protein is able to bind to p53 with the aid of E6AP and prevent p53 from inducing apoptosis by targeting it for degradation via the ubiquitin-proteasome pathway (Fig. 1).

The E6 oncoprotein is involved in two pathways associated with apoptosis (Fig. 1), including p53 inactivation and blocking apoptosis (17). Firstly, p53 inactivation may trigger the E6-induced apoptosis inhibition. p53 rapidly initiates the signaling pathway for DNA repair and apoptosis when it is activated, while the E6 proteins impair the p53-triggered signaling pathways and the cell death. In addition to the cytotoxic damage, the improper stimulation of DNA synthesis, such as HPV infection, may also activate p53 and induce apoptosis. HPVs stimulate DNA synthesis in the infected cells against the normal cell response of activating p53. The most important mechanism of p53 inactivation by high-risk HPVs is by inducing p53 degradation via the ubiquitin-proteasome pathway (18). Additionally, E6 proteins in high-risk HPV may also inhibit p53 activation by blocking the alternate reading frame p14 (p14/ARF) pathway (19), and by interacting with a histone acetyltransferase, hADA3 (20). Secondly, inhibition of apoptosis may be triggered by the E6 oncoprotein. In addition to p53-mediated apoptosis, p53-independent apoptosis is also able to eliminate abnormal cells, while E6 is capable of blocking apoptosis in cells and mice lacking p53 (21). Different stresses may trigger two major apoptotic pathways, the intrinsic and extrinsic pathway. Notably, the E6 protein is able to disturb these pathways and prevent cell death under endogenous and exogenous stress (22).

The intrinsic apoptotic pathway participates in apoptosis associated with the cell nucleus, which includes DNA damage, oxidative stress, starvation and other stimuli. These stresses, involving mitochondria and endoplasmic reticulum (ER), activate a series of pathways and change the balance between pro- and anti-apoptotic signals when the balance is upset. When the cell senses intrinsic stress, proapoptotic BH3-only proteins become activated and abrogate the function of antiapoptotic proteins. This allows for the formation of pores in the mitochondrial membrane, comprised of proapoptotic Bax or Bak, and the release of mitochondrial inner membrane proteins, including cytochrome *c*, apoptosis-inducing factor (AIF), endonuclease G, Smac/Diablo and Htr/Omi. The activation of these proteins may result in the cleavage of caspase 3 and 7, and ultimately lead to cell death. For the inhibition of HPV E6 oncoproteins, E6 is able to block the apoptotic pathway by indirect interaction with the protein Bak (23). The extrinsic apoptotic signaling pathway may be activated, as a part of the host response, and ‘death receptors’ on the cell surface may be induced by extracellular signals during HPV infection (24). The death receptors include tumor necrosis factor (TNF) receptor-1 (TNFR-1), Fas/CD95 and TNF-related apoptosis-inducing ligand (TRAIL) receptor (DR4 and DR5), and belong to the TNFR family. The receptors stimulate caspases 8 and 10, leading to the formation of the death-inducing signaling complex (DISC). The DISC also activates the downstream executioner caspases, such as caspases 3 and 7, and triggers apoptosis. In addition to the TNF pathway, it has also been shown that HPV-16 E6 is capable of inhibiting apoptosis stimulated by Fas and TRAIL pathways.

Although the extrinsic and intrinsic pathways have individual roles, the pathways are not isolated. Caspase 8 is activated during intrinsic apoptotic signaling via an amplification loop mediated by caspases 3 and 7. Similarly, mitochondrial signaling may be triggered during activation of the extrinsic cascade, via caspase 8-mediated cleavage of the BH3-only protein Bid. Therefore, the E6 protein targets intrinsic and extrinsic signaling, protecting the infected cells from multiple apoptotic stimuli and cross-activation between the two pathways. HPV-16 E6 has also been reported to either increase levels of c-Myc in E6-expressing cells or to exert no effect on c-Myc levels; thus, the overall role that c-Myc may have in E6-mediated cytoprotection has yet to be fully elucidated (25).

## 3. E5 oncoprotein

Of the HPV oncoproteins E5 is the smallest, and, for HPV-16, consists of 83 amino acids (26). The detection of this protein has proved difficult due to its extreme hydrophobicity, membrane localization and low levels of expression. Chang *et al*(27) analyzed HPV-16 E5 protein expression using immunohistochemistry. Since the high-risk HPV E5 gene is able to integrate into the human genome during malignant progression, the E5 gene is rarely detected in cervical tumors. The HPV-16/18 E5 mRNA and protein are expressed in anogenital low-grade squamous intraepithelial lesions (ISIL), which protect the infected cells from apoptosis during HPV infection. The HPV-16 E5 protein is primarily localized to the ER, but is also detected in the Golgi apparatus and nuclear membrane (28,29). By contrast, HPV-6 E5 is mainly localized to the Golgi apparatus (28) and the HPV-11 E5 protein is localized primarily in the nucleus (30). However, HPV E5 DNA synthesis may be enhanced following the transfection of HPV-16 E5 into primary human keratinocytes cultured in serum-starved medium (31). E5 protects the cells from apoptosis through two main pathways: inhibition of death receptor-mediated apoptosis and ER stress-induced apoptosis (31).

HPV E5 inhibits death receptor-mediated apoptosis in human keratinocytes. HPV E5 is capable of downregulating the total amount of Fas receptor and decreasing Fas location, as well as altering the formation of DISC induced by TRAIL; thus, E5 is able to impair Fas ligand (FasL)- and TRAIL-mediated apoptosis (32) (Fig. 1). The exogenous proteins may activate the cellular defense and disturb the ER homeostasis, so as to induce ER stress (33,34). HPV-16 E5 protein suppresses three main proteins in the ER stress pathway, including cyclooxygenase-2 (COX-2), X-box binding protein 1 (XBP-1) and inositol-requiring enzyme-1a (IRE1α) (35,36). Therefore, the E5 protein downregulates COX-2, XBP-1 and IRE1a, which is beneficial to viral persistence. However, the downregulation of ER stress response genes by HPV-16 E5 in primary genital keratinocytes suggests that the inhibition of this ER stress pathway is an event favorable to viral replication and persistence (35). In cervical cancer cells, EP4 protein may be activated by HPV-16 E5, which activates protein-kinase A. Protein kinase A is responsible for antiapoptotic effects, such as mediating ubiquitin-proteasome-mediated Bax degradation. In addition, the activated EP4 may also enhance the expression of vascular endothelial growth factor (VEGF), so as to lead to tumor immortalization in cervical carcinoma (37).

## 4. E7 oncoprotein

Oncoprotein E7 is a small acidic polypeptide composed of approximately 100 amino acids (38) that shares functional similarities with other viral oncoproteins, specifically adenovirus E1A and SV40 large T antigen (39). Ohlenschläger *et al*(40) revealed that the N-terminal domain of E7 protein is unfolded, while the C-terminal domain is a tightly packed zinc-binding fold. The N-terminus of E7 contains two conserved regions (CRs), CR1 and CR2. The E7 protein is also the major HPV oncoprotein and its expression is sufficient to immortalize primary human epithelial cells at a low frequency. The main target of E7 is retinoblastoma protein (pRb) and its associated proteins, p107 and p130. E7 oncoprotein in high-risk HPVs is necessary for viral pathogenesis and cellular transformation (8). The present review primarily discusses the modulation of apoptosis by HPV E7 oncoprotein.

The HPV-16 E7 oncoprotein induces p53-dependent and -independent apoptosis. E7 leads to antiapoptotic pRb degradation via a mechanism that involves association with and reprogramming of the cullin 2 ubiquitin ligase complex, indicating that E7 may trigger apoptosis (Fig. 1). It has been indicated that the C-terminal of the E7 protein contains a low-affinity pRb binding site, which interacts with pRb (41). p53 is also required for apoptosis in the retina of transgenic mice expressing this oncogene (42). There are also other pathways that trigger apoptosis involving the E7 protein. In the first pathway, for the low- or high-risk HPV, E7 protein activates apoptosis in NIH3T3 cells through a conserved Leu-X-Cys-X-Glu (LXCXE) motif in second chromodomain (CD2) of the E7 structure binding to the pRb. However, the binding ability of low-risk HPV E7 is ~10-fold lower than that of the high-risk HPV E7. In the second pathway, E7 and p21 form a complex which activates cathepsin B, an apoptotic mediator, in U2OS cells. In the third pathway, E7 protein initiates TRAIL and TNF-α-induced apoptosis in primary human keratinocytes (43). In the last pathway, it is likely that E7-induced apoptosis is associated with the interaction between E7 and E2F1. The complex is able to trigger E2F1-driven transcription, which contributes to increased apoptosis. Notably, HPV E7 is also associated with E2F6 and blocks its ability to be a transcriptional repressor (38). In addition, E2F1 stabilizes p53 through the induction of the p19ARF protein, which functions by binding directly to Mdm-2 and preventing p53 degradation.

The HVP E7 oncoprotein may also inhibit apoptosis and cytokine-mediated cell death depending on the cell and the viral types (38). For example, it was reported that HPV16 E7 was able to interact with and abrogate the growth-inhibitory activities of cyclin-dependent kinase inhibitor (CKI)sp21 and CKIsp27 to antagonize the activation of p53 (38). CKIsp21 and CKIsp27 have been implicated in TGF-β-mediated inhibition of growth. HPV 16 E7 is able to inactivate CKIsp21 and CKIsp27, and abrogate TGF-β-mediated growth inhibition (44). High- or low-risk HPV E7 are capable of interacting with the 600 kDa pRb-associated factor, p600 (45). The conjugation of E7 and p600 may protect detached cells from apoptosis, contributing to viral transformation (46). HPV-16 E7 protein inhibits TNF-α-mediated apoptosis in normal human fibroblasts by upregulating the expression of the inhibitor of apoptosis (IAP) protein, c-IAP2, and by a mechanism involving the suppression of caspase 8 activation. Siva-1 is a type of proapoptotic cellular factor that is capable of binding to the antiapoptotic protein Bcl-XL. The HPV-16 E7 interferes with the binding of Siva-1 and Bcl-XL, so the released Bcl-XL is able to fully exert its antiapoptotic function (47).

## 5. Interaction of the oncoproteins

The HPV full-length E2 protein is involved in activating or repressing the transcription of E6/E7. The E2 protein serves either as an activator or repressor of transcription, depending on the context of E2 binding sites within the promoter region (particularly for the P97 promoter) (48). The E6 and E7 oncoproteins determine the transformational extent of infected epithelial cells (49). The E6 and E7 genes are located in the same open reading frame (ORF) and are transcribed as a single bicistronic E6/E7 transcript from early promoter P97 [nucleotide (nt)31–97] (50). There is also an enhancer (nt7535–7862), located at the 5′ end of the P97 promoter in the long control region (LCR), which controls the expression of the E6/E7 transcript (49,50). E2 protein differentially regulates E6/E7 expression by binding to the ACCGN_4_CGGT palindromic sequence in four binding sites (E2BSs) in the LCR (such as P97 and the enhancer) (51). The modulation of the E2 protein to the E6/E7 transcription depends on its binding ability to the E2BSs genome structure (49). The binding of E2 (HPV-16 and -18) to promoter and enhancer is able to upregulate P97 activity. The activated P97 activates the transcription of E6, which binds to p53. Therefore, the interaction of E6 and P97 results in a decrease in the half-life of p53 within cells, and further inhibits apoptosis. In addition, HPV E2 protein stabilizes p53 and maintains apoptosis in HeLa cells (52). However, the expression of HPV-31 E2 in normal human epidermal keratinocytes (NHK) cells appears to destabilize p53. The E2F protein is released from pRb-E2F complexes when E7 binds to pRb. The members of the E2F family of transcription factors activate E2F1 expression, which may induce apoptosis in serum-starved cells (53). The repression of E7 transcription by E2 may reduce the level of the episomal E2F, decreasing the level of apoptosis. In SiHa cells, the expression of HPV-16 E2 protein may increase the activity of E2F (28). Furthermore, overexpression of HPV-31 E2 in NHK cells may also induce an increase in E2F1 mRNA expression (54). HPV-16 and -18 E2 proteins have been shown to activate transcription of HPV-16 E6 and E7 oncogenes (55); however, there are numerous other factors that affect the repression and activation of E6 and E7 oncoproteins. For example, the binding ability of E2 to E2BSs may be inhibited by E2BSs methylation in its 5′CpG islands (56). Moreover, the promoter and enhancer are regulated by cellular and viral proteins. Binding of octamer-binding factor 1 (Oct 1) and nuclear factor 1 (NF 1) to the LCR may disturb E2 interaction with P97, and lead to downregulation of P97 activity (57). In addition, HPVs exist as an integrated form in the infected cells, which may affect the progression of cervical cancer (58). The integrated form of HPV usually appears in the E1/E2 gene, leading to the inhibition of the E2-mediated regulation of E6/E7 oncoprotein (59). The HPV episomal form differentially methylates the P97 promoter and LCR (60). In conclusion, in order to avoid E2-induced apoptosis, the HPV genome modulates the survival of infected cells through the activities of E6 and E7 (8).

## 6. Conclusion

HPV prevents host-triggered apoptosis through p53 inactivation, apoptosis blocking, downregulation of TNF-R1 and a sustained expression of inhibitors of apoptosis. The primary mediators of the HPV-induced effects are the E5, E6 and E7 oncoproteins. With the exception of the function of blocking apoptosis, E5 protein may cooperate with E6 and E7 to immortalize cells, and play an inhibitory role in apoptosis. The studies discussed here suggest that the oncoproteins E5, E6 and E7 are important molecular targets for the prevention of the development of premalignant intraepithelial lesions and their progression to cancer.

## References

[1] Nichols AC, Palma DA, Chow W (2013). High frequency of activating PIK3CA mutations in human papillomavirus-positive orpharyngeal cancer. JAMA Otolaryngol Head Neck Surg.

[2] Yuan CH, Filippova M, Duerksen-Hughes P (2012). Modulation of apoptotic pathways by human papillomaviruses (HPV): mechanisms and implications for therapy. Viruses.

[3] Kaspersen MD, Larsen PB, Ingerslev HJ (2011). Identification of multiple HPV types on spermatozoa from human sperm donors. PLoS One.

[4] Schlecht NF, Kulaga S, Robitaille J (2001). Persistent human papillomavirus infection as a predictor of cervical intraepithelial neoplasia. JAMA.

[5] Alam MS, Ali A, Mehdi SJ (2012). HPV typing and its relation with apoptosis in cervical carcinoma from Indian population. Tumor Biol.

[6] Pisani P, Bray F, Parkin DM (2002). Estimates of the world-wide prevalence of cancer for 25 sites in the adult population. Int J Cancer.

[7] zur Hausen H (2009). Papillomaviruses in the causation of human cancers - a brief historical account. Virology.

[8] Garnett TO, Duerksen-Hughes PJ (2006). Modulation of apoptosis by human papillomavirus (HPV) oncoproteins. Arch Virol.

[9] Mischo A, Ohlenschläger O, Hortschansky P, Ramachandran R, Görlach M (2013). Structural insights into a wildtype domain of the oncoprotein E6 and its interaction with a PDZ domain. PLoS One.

[10] Cai Q, Lv L, Shao Q, Li X, Dian A (2013). Human papillomavirus early proteins and apoptosis. Arch Gynecol Obstet.

[11] Mantovani F, Banks L (2001). The human papillomavirus E6 protein and its contribution to malignant progression. Oncogene.

[12] Nominé Y, Masson M, Charbonnier S (2006). Structural and functional analysis of E6 oncoprotein: insights in the molecular pathways of human papillomavirus-mediated pathogenesis. Mol Cell.

[13] Ristriani T, Nominé Y, Masson M, Weiss E, Travé G (2001). Specific recognition of four-way DNA junctions by the C-terminal zinc-binding domain of HPV oncoprotein E6. J Mol Biol.

[14] Huibregtse JM, Scheffner M, Howley PM (1991). A cellular protein mediates association of p53 with the E6 oncoprotein of human papillomavirus type 16 or 18. EMBO J.

[15] Scheffner M, Huibregtse JM, Vierstra RD, Howley PM (1993). The HPV-16 E6 and E6-AP complex functions as a ubiquitin-protein ligase in the ubiquitination of p53. Cell.

[16] Murray-Zmijewski F, Slee EA, Lu X (2008). A complex barcode underlies the heterogeneous response of p53 to stress. Nat Rev Mol Cell Biol.

[17] Howie HL, Katzenellenbogen RA, Galloway DA (2009). Papillomavirus E6 proteins. Virology.

[18] Blanchette P, Branton PE (2009). Manipulation of the ubiquitin-proteasome pathway by small DNA tumor virus. Virology.

[19] Khoronenkova SV, Dianov GL (2011). The emerging role of Mule and ARF in the regulation of base exicision repair. FEBS Lett.

[20] Kumar A, Zhao Y, Meng G (2002). Human papillomovirus oncoperotein E6 inactivates the transcriptional coactivator human ADA3. Mol Cell Biol.

[21] Aylon Y, Oren M (2011). p53: guardian of ploidy. Mol Oncol.

[22] Contreras-Paredes A, De la Cruz-Hernández E, Martínez-Ramírez I, Dueñas-González A, Lizano M (2009). E6 variants of human papillomavirus 18 differentially modulate the protein kinase B/phosphatidylinositol 3-kinase (akt/PI3K) signaling pathway. Virology.

[23] Underbrink MP, Howie HL, Bedard KM, Koop JI, Galloway DA (2008). The E6 proteins from multiple human betapapillomavirus types degrade Bak and protect keratinocytes from apoptosis after UVB irradiation. J Virol.

[24] Gewies A (2003). Introduction to apoptosis. ApoReview.

[25] Gewin L, Galloway DA (2001). E box-dependent activation of telomerase by human papillomavirus type 16 E6 does not require induction of c-Myc. J Virol.

[26] Venuti A, Paolini F, Nasir L (2011). Papillomavirus E5: the smallest oncoprotein with many functions. Mol Cancer.

[27] Chang JL, Tsao YP, Liu DW, Huang SJ, Lee WH, Chen SL (2001). The expression of HPV-16 E5 protein in squamous neoplastic changes in the uterine cervix. J Biomed Sci.

[28] Conrad M, Bubb VJ, Schlegel R (1993). The human papillomavirus type 6 and 16 E5 proteins are membrane-associated proteins which associated with the 16-kilodalton pore-forming protein. J Virol.

[29] Borzacchiello G, Roperto F, Campo MS, Venuti A (2010). 1st international workshop on papillomavirus E5 oncogene - a report. Virology.

[30] Chen SL, Mounts P (1990). Transforming activity of E5a protein of human papillomavirus type 6 in NIH 3T3 and C127 cells. J Virol.

[31] Hu L, Potapova TA, Li S (2010). Expression of HPV16 E5 produces enlarged nuclei and polyploidy through endoreplication. Virology.

[32] Kabsch K, Alonso A (2002). The human papillomavirus type 16 E5 protein impairs TRAIL- and FasL-mediated apoptosis in HaCaT cells by different mechanisms. J Virol.

[33] Wang X, Shi Q, Xu K (2011). Familial CJD associated PrP mutants within transmembrane region induced Ctm-PrP retention in ER and triggered apoptosis by ER stress in SH-SY5Y cells. PLoS One.

[34] Xu K, Wang X, Shi Q (2011). Human prion protein mutants with deleted and inserted octarepeats undergo different pathways to trigger cell apoptosis. J Mol Neurosci.

[35] Sudarshan SR, Schlegel R, Liu XF (2010). The HPV-16 E5 protein represses expression of stress pathway genes XBP-1 and COX-2 in genital keratinocytes. Biochem Biophys Res Commun.

[36] Condjella R, Liu X, Suprynowicz F (2009). The canine papillomavirus E5 protein signals from the endoplasmic reticulum. J Virol.

[37] Oh JM, Kim SH, Lee YI (2009). Human papillomavirus E5 protein induces expression of the EP4 subtype of prostaglandin E2 receptor in cyclic AMP response element-dependent pathways in cervical cancer cells. Carcinogenesis.

[38] McLaughlin-Drubin ME, Münger K (2009). The human papillomavirus E7 oncoprotein. Virology.

[39] Toscano-Garibay JD, Benitez-Hess ML, Alvarez-Salas LM (2011). Isolation and characterization of an RNA aptamer of the HPV-16 E7 oncoprotein. Arch Med Res.

[40] Ohlenschläger O, Seiboth T, Zengerling H (2006). Solution structure of the partially folded high-risk human papillomavirus 45 oncoprotein E7. Oncogene.

[41] Liu X, Clements A, Zhao K, Marmorstein R (2006). Structure of human Papillomavirus E7 oncoprotein and its mechanism for inactivation of the retinoblastoma tumor suppressor. J Biol Chem.

[42] Ghim S, Jenson AB, Bubier JA, Silva KA, Smith RS, Sundberg JP (2008). Cataracts in transgenic mice caused by a human papillomavirus type 18 E7 oncogene driven by KRT1–14. Exp Mol Pathol.

[43] Zimmermann M, Koreck A, Meyer N (2011). TNF-like weak inducer of apoptosis (TWEAK) and TNF-alpha cooperate in the induction of keratinocyte apoptosis. J Allergy Clin Immunol.

[44] Pardali K, Moustakas A (2007). Actions of TGF-β as tumor suppressor and pro-metastatic factor in human cancer. Biochim Biophys Acta.

[45] DeMasi J, Huh KW, Nakatani Y, Münger K, Howley PM (2005). Bovine papillomavirus E7 transformation function correlates with cellular p600 protein binding. Proc Natl Acad Sci USA.

[46] DeMasi J, Chao MC, Kumar AS, Howley PM (2007). Bovine papillomavirus E7 oncoprotein inhibits anoikis. J Virol.

[47] Severino A, Abbruzzese C, Manente L (2007). Human papillomavirus-16 E7 interacts with Siva-1 and modulates apoptosis in HaCaT human immortalized keratinocytes. J Cell Physiol.

[48] Wells SI, Francis DA, Karpova AY, Dowhanick JJ, Benson JD, Howley PM (2000). Papillomavirus E2 induces senescence in HPV-positive cells via pRB- and p21(CIP)-dependent pathway. EMBO J.

[49] Mazumder Indra D, Singh RK, Mitra S, Dutta S (2011). Genetic and epigenetic changes of HPV16 in cervical cancer differentially regulate E6/E7 expression and associate with disease progression. Gynecol Oncol.

[50] Tang S, Tao M, McCoy JP, Zheng ZM (2006). The E7 oncoprotein is translated from spliced E6*I transcripts in high-risk human papillomavirus type 16-or type 18-positive cervical cancer cell lines via translation reinitiation. J Virol.

[51] Dell G, Gaston K (2001). Human papillomavirus and their role in cervical. Cell Mol Life Sci.

[52] Webster K, Parish J, Pandya M, Stern PL, Clarke AR, Gaston K (2000). The human papillomavirus (HPV) 16 E2 protein induces apoptosis in the absence of other HPV proteins and via a p53-dependent pathway. J Biol Chem.

[53] Wu X, Levine AJ (1994). p53 and E2F-1 cooperate to mediate apoptosis. Proc Natl Acad Sci USA.

[54] Frattini MG, Hurst SD, Lim HB, Swaminathan S, Laimins LA (1997). Abrogation of a mitotic checkpoint by E2 proteins from oncogenic human papillomaviruses correlates with increased turnover of the p53 tumor suppressor protein. EMBO J.

[55] Bouvard V, Storey A, Pim D, Banks L (1994). Characterization of the human papillomavirus E2 protein: evidence of trans-activation and trans-repression in cervical keratinocytes. EMBO J.

[56] Kim K, Gamer-Hamrick PA, Fisher C, Lee D, Lambert PF (2003). Methylation patterns of papillomavirus DNA, its influence on E2 function, and implications in viral infection. J Virol.

[57] Dong XP, Stubenrauch F, Beyer-Finkler E, Pfister H (1994). Prevalence of deletions of YY1-binding sites in episomal HPV 16 DNA from cervical cancers. Int J Cancer.

[58] Pett M, Coleman N (2007). Integration of high-risk human papillomavirus: a key event in cervical carcinogenesis?. J Pathol.

[59] Arisa-Pulido H, Peyton CL, Joste NE, Vargas H, Wheeler CM (2006). Human papillomavirus type 16 integration in cervical carcinoma in situ and in invasive cervical cancer. J Clin Microbiol.

[60] Bhattacharjee B, Sengupta S (2006). CpG methylation of HPV 16 LCR at E2 binding site proximal to P97 is associated with cervical cancer in presence of intact E2. Virology.

